# Mortality of Philadelphia for October, November and December, 1854; Collated from the Health Office Record; with a Summary of Deaths for the Entire Year

**Published:** 1855-02

**Authors:** Wilson Jewell


*Mortality of Philadelphia for October, November and December,* 1854; *collated from the Health Office Record; with a summary of Deaths for the entire year.* By WILSON JEWELL, M.D.

It will be noticed, by a reference to former tables, that the rate of mortality for the last three months, falls considerably below that of either of the three preceding quarters of the year.

The total of deaths for the 4th quarter, terminating December 30th, 1854, is 2,204; which number, estimating the population at 450,000, gives about 20.41 deaths in every thousand, or an average of twenty-four deaths daily—nearly one half or fifty per cent. less than for the 3d quarter of the year.

By omitting the deaths charged to Still-born, External Causes, Old Age and Unknown, amounting in all to 333, the mortality from accredited diseases alone, is only 1871, thus reducing the average of deaths from diseases to about 20 per day.

The destruction of infant life in this quarter, as in former statistics gathered from the Health Office, continues to maintain its high rate, and is of sufficient import to claim the earnest consideration and persevering investigations of every physician and political economist, in order to point out the true cause, and if removable, suggest the remedy.

Dr. Friedlander, a modern French writer, asserts that one-fourth of all children born alive, perish within the first year. Having no data of births for reference during the past three years in Philadelphia, we can only add that, in former years, the proportion ranged between one-fourth and one-fifth of all births, including, however, the still-born. Even this great sacrifice of life on its threshold, is full of interest, and ought to command the most careful study and application from the vital and political statistician.

The number of deaths recorded in the present quarter, under one year, exclusive of Still-born, amounts to 437, about one-fifth, or nearly 20 per cent. of the whole.

If a similar calculation be made of deaths under five, amounting to 973, it will exhibit a far higher rate of mortality, reaching almost to one half the whole number of deaths for the quarter.

The highest rate of mortality for any one distinct period of of life occurred under one year, viz., 437. For the next five periods, that is between 1 and 20 years, the deaths gradually diminished as the years increased. At that decade of life, between 20 and 30, the second highest rate of deaths took place, when again there was a regular declension up to the last period, 100 to 110. This arrangement is so uniform in death statistics generally, as would almost induce one to look upon it as an established law of nature.

The excess of deaths in the male sex for the quarter, has been equal to 10 per cent. over the females.

The excess of male still born children has also been 10 per cent. over those of females.

*Table No.* 2, *Class* 1*st.*—"Endemic or Epidemic diseases," amount only to 445, or 20.19 per cent. of the whole. Compared with the returns for July, August and September, it shows a reduction of 1517! This wide difference is occasioned by the falling off in the deaths from Cholera Asphyxia, Morbus and Infantum, and Dysentery and Diarrhœa.

Of Scarlet Fever there has been only six deaths in three months. In 1853, like months, there were 59 deaths.

*Class 2d.*—"Uncertain Seat;" of these there were 281 deaths. More than two-thirds were from Debility, Dropsy and Marasmus.

Nearly one-half the deaths under this head were within the first year of life.

*Class 3d.*—Of "Nervous diseases" there have been recorded 377 deaths. Of these, 129 were from convulsions, 76 of which number were under one year of age. Two hundred and twent-one, or 58·62 per cent. of all the deaths in this class were under five years of life.

*Class 4th.*—The diseases of the "Organs of Respiration" furnish 561 of the deaths for the quarter, a greater per centage than those of any other class. Of these, 349 were from Consumption of the Lungs, which in proportion to the number of deaths is as 25·83 per cent. The highest mortality from Consumption was between 20 and 30 years. From Inflammation of the Lungs there were 103 deaths.

Of the remaining classes contained in this table there is nothing remarkable. Where they present the usual variety of deaths, the numbers are unusually few.

Two hundred and twenty-two of the deaths recorded were from the Alms-house; 139 were blacks, and 12 from the country.

TABLE NO. I.

*Deaths for the fourth quarter of 1854 classified.*

| | Oct. | Nov. | Dec. | Male. O. | N. | D. | Female. O. | N. | D. | Total. |
|---|---|---|---|---|---|---|---|---|---|---|
| 1 *Endemic & Contagious diseases* Zymotic or Epidemic | 187 | 166 | 92 | 106 | 95 | 57 | 81 | 71 | 35 | 445 |
| 2 *Uncertain or general seat,* Sporadic diseases | 101 | 109 | 71 | 48 | 59 | 41 | 53 | 50 | 30 | 281 |
| 3 Nervous system | 124 | 139 | 114 | 73 | 83 | 68 | 51 | 56 | 46 | 377 |
| 4 Organs of Respiration | 176 | 191 | 198 | 74 | 91 | 107 | 102 | 100 | 91 | 565 |
| 5 " Circulation | 14 | 19 | 17 | 7 | 10 | 10 | 7 | 9 | 7 | 50 |
| 6 Digestive organs | 41 | 55 | 30 | 20 | 28 | 17 | 21 | 27 | 13 | 126 |
| 7 Urinary " | 2 | | 2 | 2 | | 2 | | | | 4 |
| 8 Organs of Generation | 1 | 5 | 4 | | | | 1 | 5 | 4 | 10 |
| 9 " Locomotion | 4 | 4 | 4 | 3 | 3 | 3 | 1 | 1 | 1 | 12 |
| 10 Integumentary system | | 1 | | | | | | 1 | | 1 |
| 11 Old age | 14 | 14 | 16 | 6 | 6 | 7 | 8 | 8 | 9 | 44 |
| 12 External causes | 32 | 35 | 25 | 28 | 22 | 18 | 4 | 13 | 7 | 92 |
| Still Born | 41 | 46 | 41 | 25 | 28 | 22 | 16 | 18 | 19 | 128 |
| Unknown | 17 | 24 | 28 | 11 | 15 | 19 | 6 | 9 | 9 | 69 |
| | 754 | 808 | 642 | 403 | 440 | 371 | 351 | 368 | 271 | 2204 |

### TABLE NO. 2.

### 1. *Endemic and Contagious Diseases—Zymotic or Epidemic.*

| | Male. | Female. | Under 1 yr. | 1 to 2. | 2 to 5. | 5 to 10. | 10 to 15. | 15 to 20. | 20 to 30. | 30 to 40. | 40 to 50. | 50 to 60. | 60 to 70. | 70 to 80. | 80 to 90. | 90 to 100. | 100 to 110. | Total. |
|---|---|---|---|---|---|---|---|---|---|---|---|---|---|---|---|---|---|---|
| Cholera Asphyxia | 34 | 22 | 1 | | 4 | 2 | 1 | 2 | 10 | 14 | 6 | 10 | 4 | 2 | | | | 56 |
| " infantum | 17 | 13 | 14 | 13 | 3 | | | | | | | | | | | | | 30 |
| " morbus | 8 | 10 | | | | 2 | 1 | 1 | 3 | 2 | 2 | 1 | 3 | 2 | 1 | | | 18 |
| Croup | 50 | 43 | 9 | 19 | 48 | 17 | | | | | | | | | | | | 93 |
| Diarrhœa | 15 | 10 | 5 | 2 | 2 | | | | 4 | 1 | 3 | 2 | 2 | 4 | | | | 25 |
| Dysentery | 33 | 26 | 2 | 9 | 6 | | | 2 | 7 | 10 | 5 | 12 | 5 | 1 | | | | 59 |
| Erysipelas | 8 | 2 | 2 | | 1 | 1 | | 1 | 1 | 3 | 1 | | | | | | | 10 |
| Fever, | 2 | 1 | 1 | | | | | | | | 1 | 1 | | | | | | 3 |
| " Bilious | 5 | | | | 2 | | | | | 1 | | 2 | | | | | | 5 |
| " Congestive | | 1 | | | | | | | | | | | | 1 | | | | 1 |
| " Intermit. | 2 | 1 | | 1 | | | | | | | | | 1 | 1 | | | | 3 |
| " Malignant | 1 | | | | | | | | 1 | | | | | | | | | 1 |
| " Remittent | 9 | 6 | 1 | 1 | 1 | 1 | | | 2 | 3 | 1 | 3 | 1 | 1 | | | | 15 |
| " Scarlet | 4 | 2 | 2 | 1 | 2 | 1 | | | | | | | | | | | | 6 |
| " Typhoid | 37 | 21 | | | 3 | 5 | | 8 | 11 | 14 | 9 | 4 | 1 | 3 | | | | 58 |
| " Typhus | 8 | 8 | | | | | | | 8 | 4 | 1 | 1 | 2 | | | | | 16 |
| Hooping Cough | 12 | 16 | 15 | 3 | 8 | 2 | | | | | | | | | | | | 28 |
| Influenza | 1 | | | | | | | | | | | | 1 | | | | | 1 |
| Measles | 2 | 1 | 1 | 1 | 1 | | | | | | | | | | | | | 3 |
| Small Pox | 9 | 3 | 2 | | 5 | | 1 | | 3 | 1 | | | | | | | | 12 |
| Syphilis | 1 | 1 | 1 | | | | | | | | | 1 | | | | | | 2 |
| | 258 | 187 | 56 | 50 | 86 | 31 | 3 | 14 | 50 | 53 | 29 | 37 | 20 | 15 | 1 | | | 445 |

### 2. *Uncertain or General Seat—Sporadic Diseases.*

| | Male. | Female. | Under 1 yr. | 1 to 2. | 2 to 5. | 5 to 10. | 10 to 15. | 15 to 20. | 20 to 30. | 30 to 40. | 40 to 50. | 50 to 60. | 60 to 70. | 70 to 80. | 80 to 90. | 90 to 100. | 100 to 110. | Total. |
|---|---|---|---|---|---|---|---|---|---|---|---|---|---|---|---|---|---|---|
| Abscess | 4 | 4 | 2 | 1 | | | | | 2 | | | 1 | 2 | | | | | 8 |
| " Glands | 1 | | | 1 | | | | | | | | | | | | | | 1 |
| " Neck | 1 | | | | | | | | | | | | 1 | | | | | 1 |
| Cancer | | 2 | | | | | | | | 1 | | | 1 | | | | | 2 |
| " Breast | | 2 | | | | | | | | 1 | | 1 | | | | | | 2 |
| Cyanosis | 7 | 7 | 13 | | | | | | 1 | | | | | | | | | 14 |
| Debility | 49 | 37 | 41 | 5 | 2 | 1 | 1 | 1 | 1 | 6 | 6 | 7 | 6 | 5 | 2 | 2 | | 86 |
| Dropsy | 13 | 18 | | 1 | 1 | 1 | | 1 | 6 | 3 | 4 | 8 | 5 | 1 | | | | 31 |
| Gangrene | 1 | | 1 | | | | | | | | | | | | | | | 1 |
| Hemorrhage | 3 | 1 | 1 | | | | | 1 | | 1 | | | 1 | | | | | 4 |
| Inanition | 15 | 6 | 18 | 1 | | | | | 1 | | | | 1 | | | | | 21 |
| Inflammation | | 1 | 1 | | | | | | | | | | | | | | | 1 |
| " parotid glands | 1 | | | | | | | | | 1 | | | | | | | | 1 |
| " throat | 1 | | 1 | | | | | | | | | | | | | | | 1 |
| Malformation | 4 | 1 | 5 | | | | | | | | | | | | | | | 5 |
| Marasmus | 39 | 40 | 43 | 20 | 9 | 1 | 1 | | 1 | 1 | | | 2 | | 1 | | | 79 |
| Mortification | | 3 | | | 1 | | | | | | | | 1 | | 1 | | | 3 |
| " Leg | 1 | | | | | | | | | | | | 1 | | | | | 1 |
| Schirrhus | | 1 | | | | | | | | 1 | | | | | | | | 1 |
| Scrofula | 1 | 6 | 4 | | 1 | | 2 | | | | | | | | | | | 7 |
| Sore Mouth | 1 | | | 1 | | | | | | | | | | | | | | 1 |
| " Throat | | 1 | 1 | | | | | | | | | | | | | | | 1 |
| Tabes Mesenterica | 4 | 2 | 4 | | | | | | | | 1 | | 1 | | | | | 6 |
| Ulceration of Face | | 1 | | | 1 | | | | | | | | | | | | | 1 |
| " Throat | 2 | | | | | 1 | | | | | | 1 | | | | | | 2 |
| | 148 | 133 | 135 | 30 | 15 | 4 | 4 | 3 | 12 | 15 | 11 | 18 | 22 | 6 | 4 | 2 | | 281 |

### 3. *Nervous Diseases.*

| | Male. | Female. | Under 1 yr. | 1 to 2. | 2 to 5. | 5 to 10. | 10 to 15. | 15 to 20. | 20 to 30. | 30 to 40. | 40 to 50. | 50 to 60. | 60 to 70. | 70 to 80. | 80 to 90. | 90 to 100. | 100 to 110. | Total. |
|---|---|---|---|---|---|---|---|---|---|---|---|---|---|---|---|---|---|---|
| Abscess of Brain | | 1 | | | | 1 | | | | | | | | | | | | 1 |
| Apoplexy | 15 | 17 | 2 | | | | | | 3 | 4 | 8 | 9 | 2 | 3 | 1 | | | 32 |
| Concussion of Brain | 3 | | | | | | 2 | | | | 1 | | | | | | | 3 |
| Congestion " | 24 | 17 | 5 | 10 | 7 | 1 | 1 | 2 | 6 | 2 | 1 | 3 | | 3 | | | | 41 |
| Convulsions | 71 | 58 | 76 | 14 | 24 | 4 | 1 | | 3 | 2 | 2 | 1 | | 2 | | | | 129 |
| " Puerperal | | 4 | | | | | | | 2 | 1 | 1 | | | | | | | 4 |
| Disease of Brain | 11 | 3 | 3 | 2 | 2 | | 3 | | 1 | 1 | 1 | | | 1 | | | | 14 |
| Dropsy " | 27 | 16 | 17 | 9 | 10 | 4 | 2 | | | 1 | | | | | | | | 43 |
| Effusion | 5 | 4 | 1 | 2 | 4 | | 1 | | | | 1 | | | | | | | 9 |
| Epilepsy | | 1 | | | | | 1 | | | | | | | | | | | 1 |
| Inflammation of Brain | 35 | 15 | 12 | 8 | 9 | 5 | 3 | 2 | 4 | 2 | | 2 | 3 | | | | | 50 |
| Insanity | | 1 | | | | | | | 1 | | | | | | | | | 1 |
| Mania | 1 | 3 | | | | | | | 1 | | 2 | 1 | | | | | | 4 |
| " a Potu | 12 | 2 | | | | | | | | 3 | 8 | 3 | | | | | | 14 |
| Neuralgia | 2 | | | | | | | | | | | | 2 | | | | | 2 |
| Palsy | 10 | 9 | | | | | | | 2 | 2 | 2 | 3 | 1 | 6 | 3 | | | 19 |
| Softening of Brain | 4 | | | | 1 | | | | | | 1 | | 1 | 1 | | | | 4 |
| Trismus | 1 | | 1 | | | | | | | | | | | | | | | 1 |
| Tetanus | 3 | 2 | 1 | 1 | | | 1 | 1 | | | 1 | | | | | | | 5 |
| | 224 | 153 | 118 | 46 | 57 | 15 | 15 | 5 | 23 | 18 | 29 | 22 | 9 | 16 | 4 | | | 377 |

### 4. *Organs of Respiration.*

| | Male. | Female. | Under 1 yr. | 1 to 2. | 2 to 5. | 5 to 10. | 10 to 15. | 15 to 20. | 20 to 30. | 30 to 40. | 40 to 50. | 50 to 60. | 60 to 70. | 70 to 80. | 80 to 90. | 90 to 100. | 100 to 110. | Total. |
|---|---|---|---|---|---|---|---|---|---|---|---|---|---|---|---|---|---|---|
| Asthma | 3 | 4 | 2 | 1 | 1 | | | | | | | | 2 | 1 | | | | 7 |
| Congestion of Lungs | 9 | 6 | 9 | 1 | 2 | | | | | 1 | | 1 | | 1 | | | | 15 |
| Consumption " | 164 | 185 | 4 | 4 | 5 | 1 | 2 | 24 | 120 | 84 | 49 | 30 | 16 | 6 | 3 | 1 | | 349 |
| Disease of Chest | 1 | | 1 | | | | | | | | | | | | | | | 1 |
| " Lungs | 5 | 5 | 2 | 1 | 1 | | | | 1 | 1 | 2 | 1 | 1 | | | | | 10 |
| Dropsy of Chest | 3 | 2 | | | | | | | 1 | 1 | | 1 | 1 | | 1 | | | 5 |
| Gangrene of Lungs | | 1 | | | | | | | | 1 | | | | | | | | 1 |
| Hectic Fever | | 1 | | | | | | | | | | | 1 | | | | | 1 |
| Hemorrhage of Lungs | 3 | 3 | | | | | 1 | 2 | 1 | | 1 | 1 | | | | | | 6 |
| Inflamm'n of Bronchiæ | 26 | 24 | 23 | 7 | 3 | | 1 | 1 | 3 | 5 | | 1 | 1 | 4 | 2 | | | 50 |
| " Chest | 1 | 1 | 1 | | 1 | | | | | | | | | | | | | 2 |
| " Larynx | 3 | 1 | 2 | 1 | | | | | | | | | | 1 | | | | 4 |
| " Lungs | 49 | 54 | 21 | 16 | 11 | 3 | 3 | 1 | 9 | 13 | 7 | 7 | 6 | 4 | 2 | | | 103 |
| " Pleura | 2 | 2 | | | | | | | 1 | 2 | | 1 | | | | | | 4 |
| " Tonsils | 1 | | | | 1 | | | | | | | | | | | | | 1 |
| Tuberculosis | 2 | 4 | | | 1 | | | 2 | 2 | 1 | | | | | | | | 6 |
| | 272 | 293 | 65 | 31 | 26 | 4 | 7 | 29 | 138 | 109 | 59 | 43 | 28 | 17 | 8 | 1 | | 565 |

5. *Organs of Circulation.*

| | Male. | Female. | Under 1 yr. | 1 to 2. | 2 to 5. | 5 to 10. | 10 to 15. | 15 to 20. | 20 to 30. | 30 to 40. | 40 to 50. | 50 to 60. | 60 to 70. | 70 to 80. | 80 to 90. | 90 to 100. | 100 to 110. | Total. |
|---|---|---|---|---|---|---|---|---|---|---|---|---|---|---|---|---|---|---|
| Anæmia | 2 | | 1 | | | | | | | 1 | | | | | | | | 2 |
| Disease of Heart | 19 | 16 | 2 | | | | 1 | 1 | 5 | 3 | 10 | 6 | 2 | 5 | | | | 35 |
| Dropsy " | 1 | 1 | | | | | | | | | | | 1 | 1 | | | | 2 |
| Enlargem't " | 2 | 1 | | | | | | | | 1 | 1 | 1 | | | | | | 3 |
| Gout " | | 1 | | | | | | | | | | | | | 1 | | | 1 |
| Inflammat'n " | | 1 | | | | | | | | | | | 1 | | | | | 1 |
| " Veins | 2 | 1 | 1 | | | | | | 2 | | | | | | | | | 3 |
| Malformation of Heart | 1 | | 1 | | | | | | | | | | | | | | | 1 |
| Ossification " | | 1 | | | | | | | 1 | | | | | | | | | 1 |
| " Arteries | | 1 | | | | | | | | | | | | | 1 | | | 1 |
| | 27 | 23 | 5 | | | | 1 | 1 | 8 | 5 | 11 | 7 | 4 | 6 | 2 | | | 50 |

6. *Digestive Organs.*

| | Male. | Female. | Under 1 yr. | 1 to 2. | 2 to 5. | 5 to 10. | 10 to 15. | 15 to 20. | 20 to 30. | 30 to 40. | 40 to 50. | 50 to 60. | 60 to 70. | 70 to 80. | 80 to 90. | 90 to 100. | 100 to 110. | Total. |
|---|---|---|---|---|---|---|---|---|---|---|---|---|---|---|---|---|---|---|
| Abscess of Liver | 2 | | | | | | | | | 1 | | 1 | | | | | | 2 |
| " Stomach | 1 | | | | | | | 1 | | | | | | | | | | 1 |
| Cancer of " | 3 | 1 | | | | | | | | | | 2 | 1 | 1 | | | | 4 |
| " Liver | 1 | 1 | | | | | | | | | | | 1 | 1 | | | | 2 |
| Cancrum Oris | 2 | 3 | | | 3 | 2 | | | | | | | | | | | | 5 |
| Cirrhosis of Liver | 1 | 1 | | | | | | | | | | 2 | | | | | | 2 |
| Congestion " | 2 | | 2 | | | | | | | | | | | | | | | 2 |
| Constipation | 1 | 1 | | | | | | | 1 | | | | 1 | | | | | 2 |
| Consumption of Bowels | 1 | | | | 1 | | | | | | | | | | | | | 1 |
| Disease of Bowels | 1 | 1 | 1 | | | | | | | | | 1 | | | | | | 2 |
| " Liver | 5 | 5 | 2 | | | | | | 1 | 1 | 1 | | 3 | 1 | 1 | | | 10 |
| " Stomach | | 2 | | | | | | | 1 | 1 | | | | | | | | 2 |
| Dropsy, Abdominal | 9 | 8 | | | | | 1 | 1 | 1 | 4 | 4 | 3 | 3 | | | | | 17 |
| Dyspepsia | 1 | | | | | | | | | | 1 | | | | | | | 1 |
| Hernia, Strangulated | 1 | 1 | | | | | 1 | | | | | 1 | | | | | | 2 |
| Inflammation of Liver | 4 | 3 | | | | | | | 1 | | 2 | 3 | | 1 | | | | 7 |
| " Peritoneum | 6 | 5 | | | | 1 | 1 | 1 | 3 | 2 | 1 | 1 | | 1 | | | | 11 |
| " Stom. & Bowels | 14 | 16 | 9 | 2 | 1 | 2 | 1 | | 2 | 2 | 2 | 2 | 1 | 6 | | | | 30 |
| Jaundice | 3 | 6 | 3 | | | | | | 2 | 1 | | 1 | 1 | 1 | | | | 9 |
| Obstruction of Bowels | 2 | 1 | | | | | | 1 | 1 | | | | 1 | | | | | 3 |
| Schirrus Pyloris | | 1 | | | | | | | | | | | 1 | | | | | 1 |
| Teething | 2 | 2 | 2 | 2 | | | | | | | | | | | | | | 4 |
| Ulceration of Intestines | 1 | 3 | | | | 2 | | 1 | | | | 1 | | | | | | 4 |
| " Rectum | 1 | | | | | | | | | | | 1 | | | | | | 1 |
| Worms | 1 | | | 1 | | | | | | | | | | | | | | 1 |
| | 65 | 61 | 19 | 5 | 5 | 7 | 4 | 5 | 13 | 12 | 11 | 19 | 13 | 12 | 1 | | | 126 |

7. *Urinary Organs.*

| | Male. | Female. | Under 1 yr. | 1 to 2. | 2 to 5. | 5 to 10. | 10 to 15 | 15 to 20. | 20 to 30. | 30 to 40. | 40 to 50. | 50 to 60. | 60 to 70. | 70 to 80. | 80 to 90. | 90 to 100. | 100 to 110. | Total. |
|---|---|---|---|---|---|---|---|---|---|---|---|---|---|---|---|---|---|---|
| Abscess of Kidneys, | 1 | | | | | | | | | 1 | | | | | | | | 1 |
| Disease " | 1 | | | | | | | | | | 1 | | | | | | | 1 |
| Inflammation " | 1 | | | | | 1 | | | | | | | | | | | | 1 |
| " Urinary Organs | 1 | | | | | | | | | | | | 1 | | | | | 1 |
| | 4 | | | | | 1 | | | | 1 | 1 | | 1 | | | | | 4 |

8. *Organs of Generation.*

| | Male. | Female. | Under 1 yr. | 1 to 2. | 2 to 5. | 5 to 10. | 10 to 15. | 15 to 20. | 20 to 30. | 30 to 40. | 40 to 50. | 50 to 60. | 60 to 70. | 70 to 80. | 80 to 90. | 90 to 100. | 100 to 110. | Total. |
|---|---|---|---|---|---|---|---|---|---|---|---|---|---|---|---|---|---|---|
| Cancer of Uterus | | 4 | | | | | | | 1 | 1 | 2 | | | | | | | 4 |
| Child-bed, | | 2 | | | | | | | 1 | 1 | | | | | | | | 2 |
| Inflammation of Uterus | | 1 | | | | | | | | 1 | | | | | | | | 1 |
| Puerperal Fever | | 1 | | | | | | | 1 | | | | | | | | | 1 |
| Rupture of Uterus | | 1 | | | | | | | | 1 | | | | | | | | 1 |
| Ulceration " | | 1 | | | | | | | | | | | 1 | | | | | 1 |
| | | 10 | | | | | | | 3 | 4 | 2 | | 1 | | | | | 10 |

9. *Organs of Locomotion.*

| | Male. | Female. | Under 1 yr. | 1 to 2. | 2 to 5. | 5 to 10. | 10 to 15. | 15 to 20. | 20 to 30. | 30 to 40. | 40 to 50. | 50 to 60. | 60 to 70. | 70 to 80. | 80 to 90. | 90 to 100. | 100 to 110. | Total. |
|---|---|---|---|---|---|---|---|---|---|---|---|---|---|---|---|---|---|---|
| Caries of Spine | 1 | | | | | | 1 | | | | | | | | | | | 1 |
| Disease " | 3 | 1 | | | | | 1 | | 1 | | 1 | | | 1 | | | | 4 |
| " of Hip Joint | 2 | 1 | | | | | 1 | 2 | | | | | | | | | | 3 |
| Rheumatism | 2 | 1 | | | | | | | 1 | | | 2 | | | | | | 3 |
| White Swelling | 1 | | | | | | | | 1 | | | | | | | | | 1 |
| | 9 | 3 | | | | | 3 | 2 | 3 | | 1 | 2 | | 1 | | | | 12 |

10. *Integumentary System.*

| | Male. | Female. | Under 1 yr. | 1 to 2. | 2 to 5. | 5 to 10. | 10 to 15. | 15 to 20. | 20 to 30. | 30 to 40. | 40 to 50. | 50 to 60. | 60 to 70. | 70 to 80. | 80 to 90. | 90 to 100. | 100 to 110. | Total. |
|---|---|---|---|---|---|---|---|---|---|---|---|---|---|---|---|---|---|---|
| Purpura Hemorrhagica | | 1 | 1 | | | | | | | | | | | | | | | 1 |

11. *Old Age.*

| | Male. | Female. | Under 1 yr. | 1 to 2. | 2 to 5. | 5 to 10. | 10 to 15. | 15 to 20. | 20 to 30. | 30 to 40. | 40 to 50. | 50 to 60. | 60 to 70. | 70 to 80. | 80 to 90. | 90 to 100. | 100 to 110. | Total. |
|---|---|---|---|---|---|---|---|---|---|---|---|---|---|---|---|---|---|---|
| Old Age | 19 | 25 | | | | | | | | | | | 6 | 15 | 13 | 9 | 1 | 44 |

12. *External Causes.*

| | Male. | Female. | Under 1 yr. | 1 to 2. | 2 to 5. | 5 to 10. | 10 to 15. | 15 to 20. | 20 to 30. | 30 to 40. | 40 to 50. | 50 to 60. | 60 to 70. | 70 to 80. | 80 to 90. | 90 to 100. | 100 to 110. | Total. |
|---|---|---|---|---|---|---|---|---|---|---|---|---|---|---|---|---|---|---|
| Asphyxia | 1 | 2 | 3 | | | | | | | | | | | | | | | 3 |
| Burns | 8 | 3 | 1 | 1 | 4 | 1 | | | 2 | | 1 | 1 | | | | | | 11 |
| Casualties | 22 | 3 | 2 | | 1 | | 4 | 1 | 7 | 7 | 3 | | | | | | | 25 |
| Drowned | 17 | 2 | | | 2 | | | 1 | 3 | 5 | 3 | 1 | 3 | 1 | | | | 19 |
| Fracture of Ankle | 1 | | | | | | | | 1 | | | | | | | | | 1 |
| " Leg | 1 | | | | | | | | | 1 | | | | | | | | 1 |
| " Skull | 1 | | | | | | | | | 1 | | | | | | | | 1 |
| Intemperance | 8 | 8 | | | | | | | 3 | 2 | 7 | 1 | 1 | 2 | | | | 16 |
| Poison | 2 | 1 | | | | | | 1 | | 1 | 1 | | | | | | | 3 |
| Suffocation | | 4 | 3 | | | | | 1 | | | | | | | | | | 4 |
| Suicide | 6 | | | | | | | 1 | | 1 | 2 | 1 | | 1 | | | | 6 |
| Violence | 1 | 1 | 2 | | | | | | | | | | | | | | | 2 |
| | 68 | 24 | 11 | 1 | 7 | 1 | 4 | 5 | 16 | 18 | 17 | 4 | 4 | 4 | | | | 92 |
| Still Born | 75 | 53 | 128 | | | | | | | | | | | | | | | 128 |
| Unknown | 45 | 24 | 27 | 1 | 6 | | 1 | 1 | 6 | 4 | 11 | 5 | 5 | 1 | 1 | | | 69 |

### TABLE NO. 3.

*Deaths for the Fourth Quarter of* 1854, *at fifteen distinct periods of life.*

| | |
|---|---|
| Under 1 year, | 437 |
| 1 to 2 | 164 |
| 2 to 5 | 202 |
| 5 to 10 | 63 |
| 10 to 15 | 42 |
| 15 to 20 | 65 |
| 20 to 30 | 272 |
| 30 to 40 | 239 |
| 40 to 50 | 182 |
| 50 to 60 | 157 |
| 60 to 70 | 113 |
| 70 to 80 | 93 |
| 80 to 90 | 34 |
| 90 to 100 | 12 |
| 100 to 110 | 1 |
| | 2076 |
| Still Born | 128 |
| Total, | 2204 |

Included within the above table, were 222 from the Blockley Alms House; 139 Blacks, and 12 from the country, as follows:

| | October. | November. | December. | Total. |
|---|---|---|---|---|
| Almshouse, | 82 | 77 | 63 | 222 |
| Blacks, | 53 | 42 | 44 | 139 |
| Country, | 4 | 4 | 4 | 12 |
| Total, | | | | 373 |

### Annual Mortality of Philadelphia, 1854.

By comparing the mortality of 1854 with that of 1853, the most notable difference is the increase of the former over the latter. This, however, to some extent, at least, may be accounted for, aside from an epidemic influence, by the additional amount of territory with its sub-urban population engrafted on the city proper and its incorporated districts, from which alone, before the act of consolidation, the mortuary tables were calculated. Now, the returns from a number of burial grounds scattered over the county, are added to the former list, and instead of calculating the death rates from the returns of a population estimated at 415,000, we now embrace the population of the entire county, and from the most reliable evidence to be obtained, venture to set it down at 450,000.

Starting with this proposition, the relative numerical difference of mortality between these two years may be explained away, especially when taken together, with the redundancy of deaths which marked the presence of the several varieties of Cholera as well as Endemic Dysentery, during the past summer.

During the fifty-two weeks embraced within the dates January 1st and December 30th, 1854, there have died of the population of our city 11,784. By reducing to a mean, the population for the year, which gives 432,500, it follows that the average of deaths would be 36·70 in every thousand, or 32·28 for each day, 226.1-7 each week, and 982 each month.

The increase over the deaths for 1853, which amounted to 9,744, has been 2,040, equivalent to 9·48 per cent.

Of the total number of deaths, 3,309, or 28·08 per cent. perished within the first year of life. Five thousand eight hundred and fifty-three, or 49·58 per cent. within the fifth year. Under twenty, 6,800, or 58·70 per cent. These calculations include the Still-born.

Only fifty-three of all who died, lived until the tenth decade of life—over 90—and 13 were centennarians.

The following table will exhibit at a glance the mortality for the year, of diseases grouped together in classes, and from other causes, together with the rate of per centage compared with the whole number of deaths:

| | | Yearly Mortality. | Per cent. to whole number. |
|---|---|---|---|
| 1. | Endemic and Contagious diseases. | | |
| | Zymotic or Epidemic, | 3306 | 28·05 |
| 2. | Uncertain or General Seat. | | |
| | Sporadic diseases, | 1375 | 11·66 |
| 3. | Nervous System, | 2176 | 18·46 |
| 4. | Organs of Respiration, | 2429 | 20·61 |
| 5. | " Circulation, | 241 | 2·04 |
| 6. | " Digestion, | 585 | 4·96 |
| 7. | Urinary Organs, | 34 | 0·28 |
| 8. | Organs of Generation, | 79 | 0·66 |
| 9. | " Locomotion, | 47 | 0·39 |
| 10. | Integumentary System, | 15 | 0·12 |
| 11. | Old Age, | 196 | 1·66 |
| 12. | External Causes, | 446 | 3·77 |
| | Still Born, | 534 | 4·44 |
| | Unknown, | 321 | 2·87 |

From this analysis, we learn that the Endemic and Epidemic diseases, amounting to 3,306, presented the highest rate of mortality during the year, equal to 28·05 per cent. The organs of Respiration, the next highest rate, 2,429, being equivalent to 20·61 per cent. of the whole.

Still-born children give 534, or 4·44 per cent. of the deaths, while Old Age furnishes but 196, or 1·66 per cent.

The ratio of deaths in each sex, according to the months of the year, was as follows:

| Months. | Males. | Females. | Total. |
|---|---|---|---|
| January | 387 | 362 | 749 |
| February | 547 | 477 | 1024 |
| March | 435 | 372 | 807 |
| April | 407 | 365 | 771 |
| May | 457 | 403 | 860 |
| June | 453 | 384 | 838 |
| July | 985 | 805 | 1790 |
| August | 939 | 832 | 1771 |
| September | 526 | 444 | 970 |
| October | 403 | 351 | 754 |
| November | 440 | 368 | 808 |
| December | 371 | 271 | 612 |
| | 6350 | 5434 | 11784 |

According to this exhibition, there were 6,350 male deaths and 5,434 female deaths; showing an excess of 7·77 per cent. of the former over the latter.

The highest mortality took place in July, viz., 1790, presenting an average of 58 deaths per day. The next highest in August, or 57 per day; then February, or 36 per day. Then follow in order September, May, June, November, March, April, October, January, December, which last is the lowest, 642, or 20 deaths per day.

Consumption of the Lungs has supplied by far the greatest outlet to human life during the year, numbering 1,394, equivalent to 11·82 per cent. of the whole number of deaths, less 1 per cent. than those for 1853, compared with the total of deaths for the year.

All ages have succumbed to the power of this disease. Thirty-two were in earliest infancy, within the first year of life. Between the ages of twenty and thirty, 438, and between thirty and forty, 306, amounting to more than half of all that occurred. One was over 90, and one beyond one hundred years of age. The excess of the deaths was among females, equal to 3·29 per cent.

During the year the Alms-house furnished 951, or 8·07 per cent. of the whole number of deaths. The colored population 709, or 6·01 per cent, while there were 67 brought in from the country to add a fraction to the mortality of the city.

The subjoined table presents a synopsis of the diseases which have been most fatal, and hence most prevalent, for seven consecutive years in this city:

| | 1848 | 1849 | 1850 | 1851 | 1852 | 1853 | 1854 |
|---|---|---|---|---|---|---|---|
| Cholera Infantum | 454 | 582 | 505 | 397 | 329 | 398 | 715 |
| Consumption of Lungs | 965 | 939 | 907 | 881 | 1204 | 1246 | 1394 |
| Congestion of Brain | 85 | 91 | 97 | 130 | 120 | 172 | 222 |
| Convulsions | 401 | 415 | 444 | 479 | 499 | 543 | 695 |
| Croup | 177 | 130 | 143 | 180 | 208 | 303 | 304 |
| Diarrhœa | 122 | 225 | 208 | 157 | 156 | 130 | 219 |
| Dropsy of the Brain | 220 | 237 | 283 | 245 | 247 | 192 | 299 |
| Dysentery | 315 | 578 | 421 | 401 | 558 | 379 | 446 |
| Inflammation of Brain | 186 | 198 | 218 | 202 | 258 | 157 | 362 |
| " Bronchiæ | 172 | 169 | 191 | 175 | 208 | 197 | 243 |
| " Lungs | 265 | 273 | 352 | 352 | 444 | 353 | 472 |
| Marasmus | 237 | 264 | 217 | 255 | 354 | 376 | 457 |
| Scarlet Fever | 172 | 242 | 439 | 400 | 433 | 391 | 165 |
| Small Pox | 100 | 152 | 40 | 216 | 426 | 52 | 40 |

To the sanitarian and the medico-politician, as well as to every philanthropist, it becomes an interesting subject of inquiry, what proportion of the annual deaths of our city for 1854, or any other past year, have been occasioned by untimely and preventable diseases. To arrive at correct conclusions on this important topic, we need scarcely advert to the necessity of appealing to the average deaths of a series of years. To illustrate the subject by the mortality of an isolated period, would furnish only a rational approximation to the truth; yet enough may be gathered from the estimate, to throw some light upon the great disproportion of those deaths in the midst of an urban population, preventable and premature, to those which are the result of natural causes or the fixed laws of nature.

Acute diseases of almost every form, dependant in a great measure on local and obviable causes, rob our population annually of thousands in early infancy, in the vigor of youth and in the prime of manhood; and be it known, that it is scarcely reserved for a twentieth part of our community to reach its "three score years and ten."

This early abridgment of human life, this rapid decay, this desecration of the living, would seem to be peculiar to metropolitan populations, and if the records of those historians who have supplied us with documentary evidence, and traced from year to year the effects of the large expenditure of life in cities to their causes, may be relied upon, then may we naturally conclude that the remedy lies within our reach—an efficient sanitary organization—which, in its widely adapted signification, shall apply its scientific labors to the physical, social and, we would add, the moral condition of a large proportion of our population.

We need not, however, enlarge on this vital and indispensable department of sanitary science, but pass to consider the great pressure of mortality on the young in our city as the result of preventable diseases, and the large disproportion between them and those more advanced in life.

As a single illustration of the large demand made by death upon our infant population, we need only advert to the fact that, during the year 1854, five thousand three hundred and nineteen

(5319) children died in the first five years of life,* equal to 47·28 per cent of the whole number of deaths, while, according to the census of 1850, the proportion of our population under five years is not over 1·04 per cent.

This enormous extinction of life in early infancy, as compared with the proportion of children to the whole population, must depend upon certain physical causes, which, when brought under an efficient sanitary organization, would in all probability prove comparatively inert.

If we look carefully over that class of acute diseases enrolled under the head of "Epidemic or Endemic," many of which owe their fatality to local and remediable influences, we shall find that, of the whole number of deaths for the year, amounting to 3,306, nineteen hundred and ninety-nine (1,999) or 60·49 per cent., were among children under five years of age.

Nearly the same rate prevails in some other classes, and it is not irrational to infer, that if a judicious attention should be directed towards the remedying of those physical, social and moral evils which contribute a very heavy per centage in the destruction of infantile life in large cities, the mortality would be reduced one half its present average.

Of the whole number of deaths for the year 1854, it would not be unreasonable to contend, that two-fifths of them owe their occurrence and are, perhaps, intimately dependent for their origin on causes which are easily recognized as local and removable.

By separating those deaths in the table which come under the head of Still-born, Old Age, Unknown, External Causes and Debility, amounting in the aggregate to 1,955, and estimating that one-half the remainder are the result of chronic diseases, which do not come within the sphere of preventable deaths, we have left 4,914 properly belonging to acute ailments, which under an improved system of sanitary science, are partially or wholly capable of being so far ameliorated as to be deprived of their fatal character.

* This calculation does not include the Still-born children, which amounted to 534.

Of that distinction of deaths regarded as the result of diseases local and preventable in their character, may be included the Epidemic and Endemic or Zymotic, Marasmus, Convulsions and Diseases of the Brain in Children, Inflammation of the Lungs and Bronchiæ, acute diseases of the Liver, Stomach and Bowels, Intemperance and Mania-a-potu.

Valuable indeed will be the reform in our sanitary police arrangements, when the death rate is reduced to one-half its present per centage; but while there is no likelihood of so pleasing an immunity from disease and death in our city as the above suggestion contemplates, we may still dwell on the promise of reform from year to year, as the science of Hygiene advances, until the population of all large cities shall compare in health with rural districts.

